# Regards to the History of Neurosurgery Malaysia: Past, Present and Future

**DOI:** 10.21315/mjms2022.29.6.19

**Published:** 2022-12-22

**Authors:** Muhammad Ihfaz Ismail, Song Yee Ang, Diana Noma Fitzrol

**Affiliations:** 1 Brain and Behaviour Cluster (BBC), Department of Neurosciences, School of Medical Sciences, Universiti Sains Malaysia, Kelantan, Malaysia; 2 Hospital Universiti Sains Malaysia, Universiti Sains Malaysia, Kelantan, Malaysia

Dear Editor,

I read with interest the article *History of neurosurgery in Malaysia: The past, present and future* (1). We would like to highlight the history, present and the future of neurosurgery services at Hospital Universiti Sains Malaysia (USM). Beginning as the Unit of Neurosurgery under the Department of General Surgery, the Department of Neurosciences was established as an independent department headed by Dato’ Professor Dr Jafri Malin Abdullah since he came back from Belgium from 1995 until 2015. He was succeeded by Professor Dr Zamzuri Idris. Six neurosurgeons teach neurosurgical sciences and other USM programmes (Figure 1).

The Department of Neurosciences of the School of Medical Sciences is unique and comprehensive since it includes neurosurgeons, neurologists, neuro-anaesthetists, neurorehabilitation specialists and neuroscientists. It provides comprehensive neurological and neurosurgical services to the east Coast of Peninsular Malaysia specifically and to the whole of Malaysia generally. These responsibilities and achievements are made possible by the availability of up-to-date facilities for patient services and by ensuring concurrent high-quality teaching and research for medical, surgical, and other allied health trainees.

The department’s diagnostic facilities include a 128 slice CT scanner, a 3 Tesla functional MRI, magnetoencephalography, an angiography suite and electrophysiological studies. The centre is well-equipped with transcranial magnetic stimulation facilities and complete behaviour, neurogenetics, rodent animal behaviour, stem cell culture and primary brain cell culture laboratories. Professor Dr Zamzuri places equal emphasis and focus on the advancement of neuroscience fields such as genomic, proteomic and metabolics studies.

The department is the main centre involved in the National Training Centre for Neurosurgery in addition to the Ministry of Health Malaysia hospitals and the other university hospitals that recently joined—Universiti Malaya (UM) and Universiti Kebangsaan Malaysia (UKM). For training and healthcare purposes, elective surgery is conducted at least three times a week, while an emergency operation theatre is available every day. Patients are nursed in a 12-bed intensive care and high dependency unit. The department has a complete brain and spine frameless radiosurgery system and a Zeiss photon beam intraoperative radiotherapy system in an interventional radiotherapy operative suite. Image-guided systems are used for surgery and rehabilitation.

The centre offers general neurosurgery, neuro-oncology, vascular neurosurgery, peripheral nerve surgery, functional neurosurgery and paediatric neurosurgery. Under the excellent leadership of Professor Dr Zamzuri Idris, the department is preparing to become a subspeciality training centre for functional deep brain stimulation (DBS) (Figure 2), anterior skull base surgery (endoscopic transphenoidal and open), lateral skull base surgery (retrosigmoid, translabyrinthine and transpetrosal approaches), epilepsy surgery and vascular surgery for complex cerebrovascular diseases.

The National Training Programme for the Master of Neurosurgery consists of two years of surgical rotation plus four years of focused, comprehensive clinical training aimed at developing competent neurosurgeons who can provide effective, safe services, fulfilling the country’s needs. The Department of Neurosciences, under the excellent leadership of Professor Dato’ Dr Jafri Malin Abdullah and Professor Dr Zamzuri Idris, has been involved in teaching and graduating more than 110 neurosurgeons via the Master of Surgery (Neurosurgery). With the establishment of this local training programme, neurosurgery services have been further extended to more states, including Hospital Sultanah Bahiyah, Kedah, Hospital Tunku Ampuan Afzan Kuantan, Pahang, Hospital Sultanah Nur Zahirah Terengganu and Hospital Tuanku Jaafar Seremban Negeri Sembilan, in addition to Hospital Sultanah Aminah (HSA) Johor Bahru, Institute Kaji Saraf Tunku Abdul Rahman (IKTAR) Hospital Kuala Lumpur (HKL), Penang General Hospital, Sungai Buloh Hospital and Sarawak General Hospital and Sabah General Hospital.

Until 2020, Universiti Sains Malaysia was the first and only local training programme for neurosurgery, which was established in 2001 under the guidance and leadership of Professor Dato’ Dr Jafri Malin Abdullah in the Department of Neurosciences, School of Medical Sciences Universiti Sains Malaysia. Subsequently, the Universiti Malaya was established as a second national training centre for Neurosurgery in Malaysia, whereby three students were enrolled in this university as the first students for the Master of Neurosurgery on 1 December 2020.

Apart from becoming the centre of national training for the Master of Neurosurgery, the Department of Neurosciences is actively involved in brain behaviour cluster (BBC) activities placed under the School of Medical Sciences, Universiti Sains Malaysia Kubang Kerian Kelantan, under the chairman Professor Dato’ Dr Jafri Malin Abdullah (2). This cluster combines several schools and departments from the main, health and engineering campuses, and the Advanced Medical and Dental Institute. The cluster’s main purpose is to create clinical transdisciplinary services and conduct research. The BBC continues the Centre for Neuroscience Services and Research (P3Neuro), which was formally established on 22 December 2015 to provide transdisciplinary services and research, with larger clinical combinations and multiple disciplines with broader objectives.

In 2013, which was 9 years ago, Professor Dato’ Dr Jafri Malin Abdullah initiated Asia’s first Integrated Neurosciences Programme, consisting of the Master of Neurosciences, Doctor of Neurosciences, Master of Cognitive Neurosciences, and an integrated Master of Psychology (Clinical), Doctor of Psychology (Clinical Psychology) and Doctor of Psychology (Clinical Neuropsychology), and Master of Cognitive Neurosciences. Figures 3–14 depict the current students studying at the USM in the Master of Surgery (Neurosurgery), Master of Cognitive Neurosciences and Master and Doctor of Psychology and Master of Integrated Neuroscinece Programme. A total of 123 neuroscientists, including clinical psychologists, have graduated and provided services to the entire nation.

The Neurosurgical Association of Malaysia recently held its 20th annual scientific meeting in conjunction with the Penang International Neurosurgical Congress from 25–27 August 2022. This special event was officiated by Tun Yang Terutama (TYT) Tun Dato’ Seri Utama Ahmad Fuzi bin Haji Abdul Razak, the Governor of Pulau Pinang (Figure 15). At the meeting, TYT Tun launched the book *History of neurosurgery in Malaysia* by YBhg Dato’ Dr Fadli Cheah Abdullah (Figure 16).

## Figures and Tables

**Figure 1:**
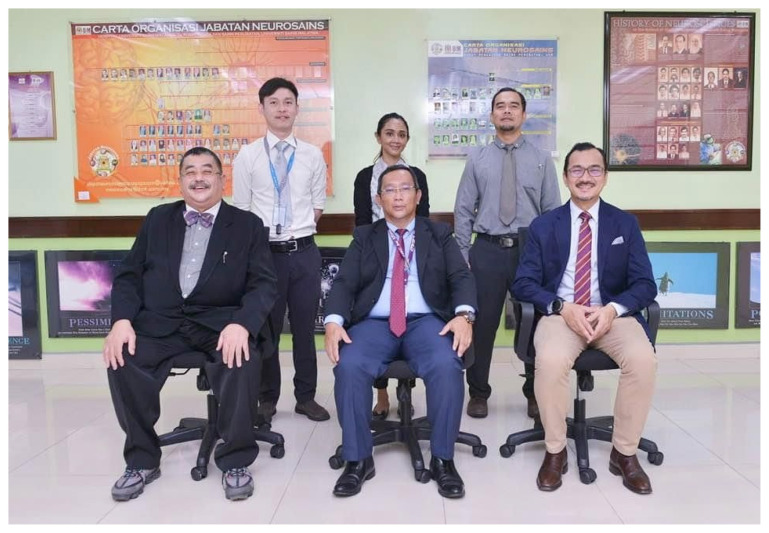
Back (L–R): Dr Ang Soon Yee, Dr Diana Fitzrol and Dr Muhammad Ihfaz Ismail. Front (L–R): Professor Dato’ Dr Jafri Malin Abdullah, Professor Dr Zamzuri Idris and Professor Dato’ Dr Abdul Rahman Izaini Ghani

**Figure 2:**
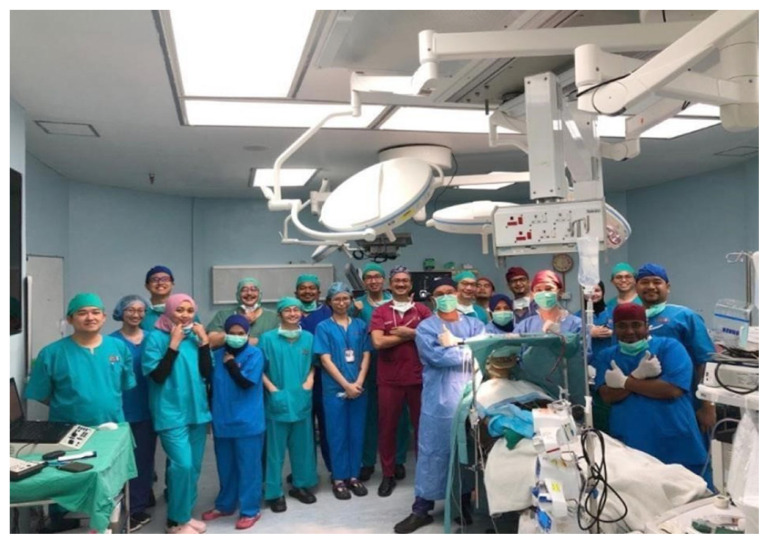
Awake surgery for DBS, Hospital USM, 22 June 2022. Picture taken after both electrodes were inserted. The surgery was led by Professor Dato’ Dr Abdul Rahman Izaini Ghani, Professor Dato’ Dr Jafri Malin Abdullah and Professor Dr Zamzuri Idris, who were joined by a team of neurosurgeons, neurologists and neuro-anaesthesists from Hospital Tuanku Jaafar Seremban, Negeri Sembilan, as their initial step before setting up a DBS programme in their hospital

**Figure 3:**
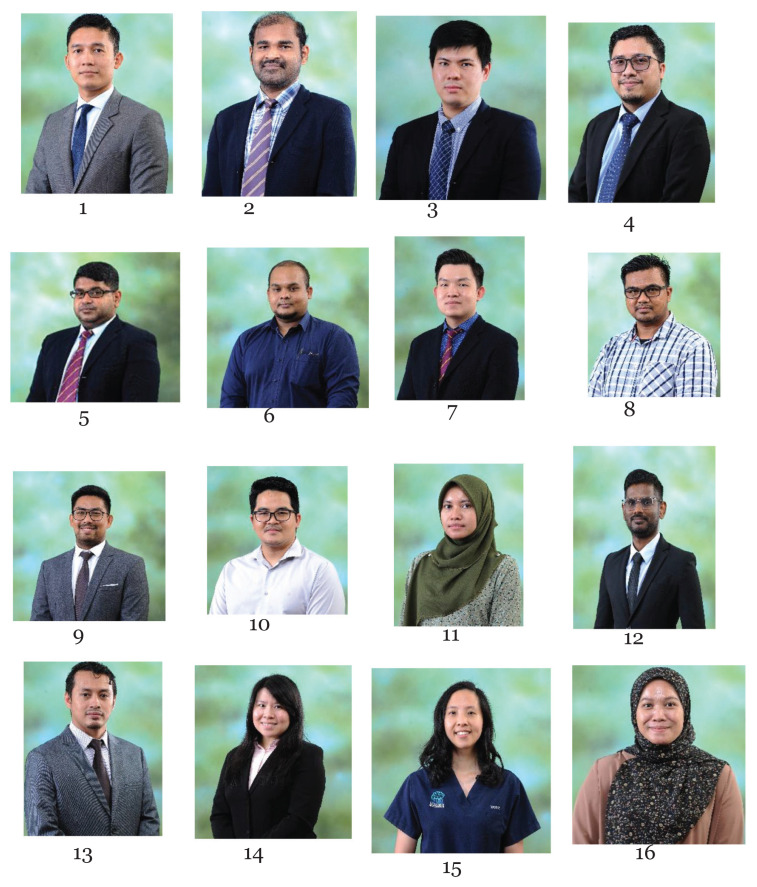
Master of Neurosurgery Year 1 students (November 2022 intake): (1) Dr Alif Faliq Iskandar bin Zulkifli, (2) Dr Balamurugan a/l Rajendran, (3) Dr Eddie Lim Wei Ming, (4) Dr Harivarmah a/l Nagalinggam, (5) Dr Kalaishaan a/l Raman, (6) Dr Karrthik a/l Murugasan, (7) Dr Lee Calwin, (8) Dr Mohamad Lokman bin Abdul Aziz, (9) Dr Muhamad Syafik bin Abdul Aziz, (10) Dr Muhammad Asyraf bin Muhamad Yunos, (11) Dr Nadhira binti Mhd Razali, (12) Dr Sivakumaran Rajandran, (13) Dr Syamsul Anwar bin Kamarudin, (14) Dr Tan Hui Khing, (15) Dr Woo Xiangling and (16) Dr Rahimah binti Ismail

**Figure 4:**
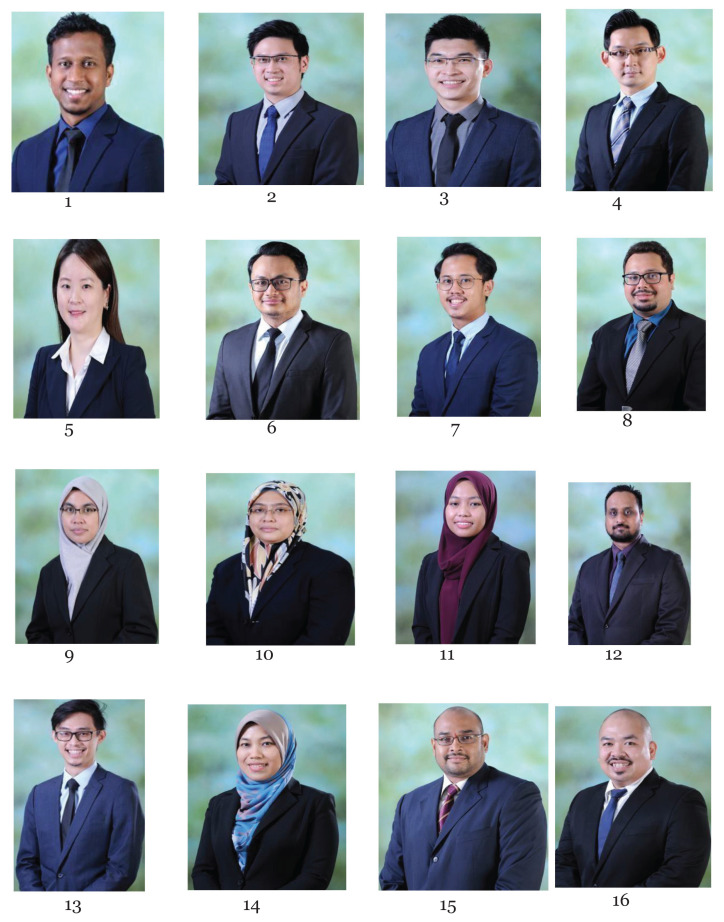
Master of Neurosurgery Year 1 students (December 2021 intake): (1) Dr Anantha Kumar a/l Vadiveloo, (2) Dr Ho Yan Zheng, (3) Dr Jonathan Wong Kee Chi, (4) Dr Lee Wei Lun, (5) Dr Lim Xiao Yi, (6) Dr Mohamad Hidir Abdullah, (7) Dr Mohd Fakhri Mohd Fathil, (8) Dr Mokk Kanapathy Pillay a/l Kasinathan, (9) Dr Murni Mohamed Fuad, (10) Dr Nur Izzah Izni Ghazali, (11) Dr Nurul Ashikin Hamzah, (12) Dr Suriaraja a/l Guvindan Raju, (13) Dr Tan Guan Yan, (14) Dr Umaira Saleh, (15) Dr Vasu a/l Nallaluthan and (16) Dr Zhafran Mohammad Razif

**Figure 5:**
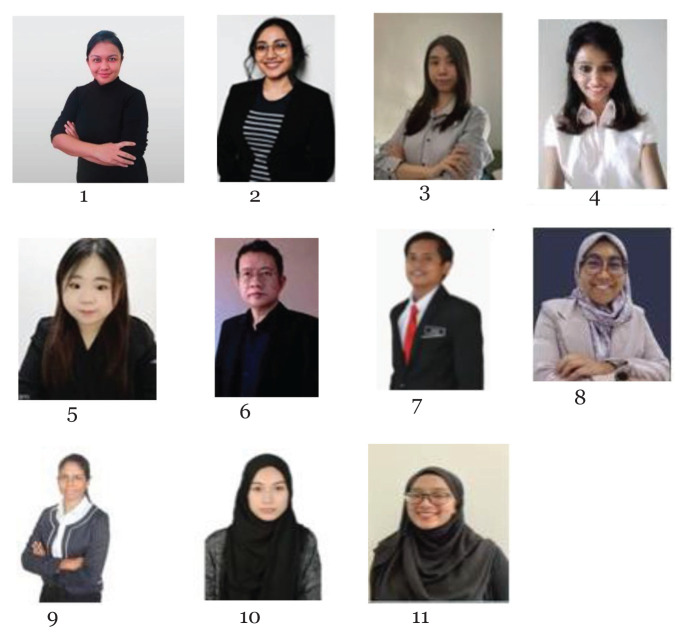
Master of Cognitive students (7th batch): (1) Ainun Aida binti Bahardin, (2) Annalia Rashna Banun Anak Ivanhoe Anthony, (3) Gong Yiyun, (4) Kouseleya a/p Vasuthevan, (5) Loo Heoy Min, (6) Lee Meng Siong, (7) Mohamad Nur Al Hakim bin Hamdan, (8) Nur Farah Farhana binti Jamil, (9) Rajaeswary a/p Thangarasah, (10) Sharifah Zahrah Alwi Alkaf and (11) Tunishah binti Jayaprakash

**Figure 6:**
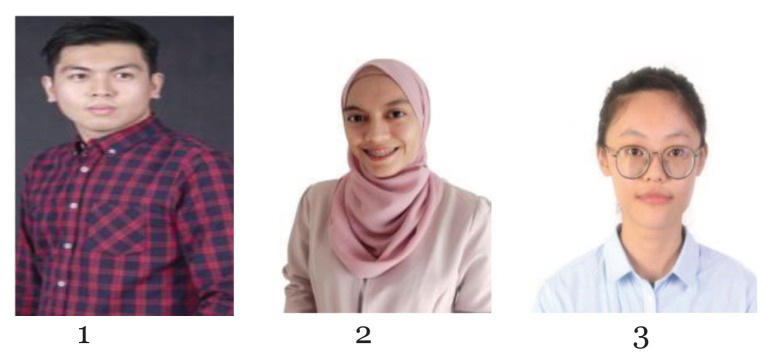
Master of Cognitive Neurosciences students (8th batch): (1) Tedd bin Denis, (2) Nisa Athirah binti Zahadi and (3) Ng Shing Sian

**Figure 7:**
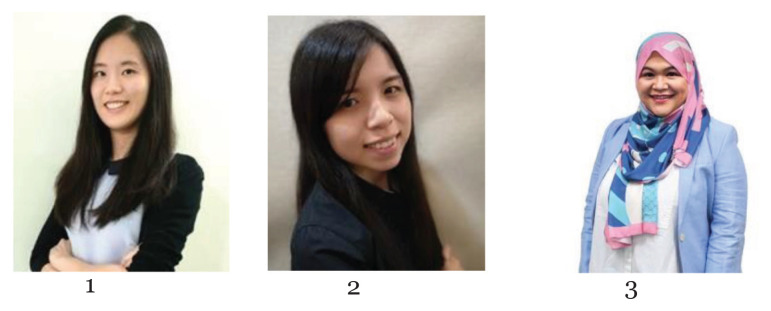
Doctor of Psychology (Clinical Psychology) students (2nd batch): (1) Lee Sook Huey (UPSI), (2) Melody Chee Hui Ni (USM) and (3) Putri Intan Dianah binti Megat Burhainuddin (USM)

**Figure 8:**
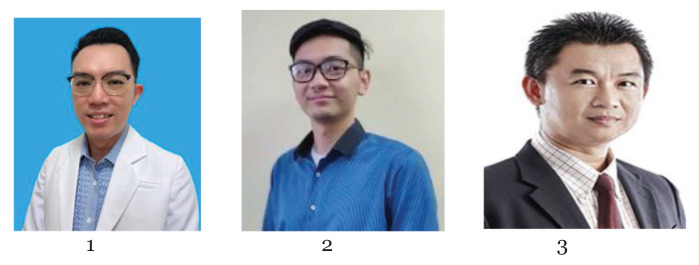
Doctor of Psychology (Clinical Neuropsychology) students (1st batch): (1) Mah Siew Chung (USM), (2) Mohamad Farahan Huszaimi M. Pajar (USM) and (3) Ng Wei Song (UPSI)

**Figure 9:**
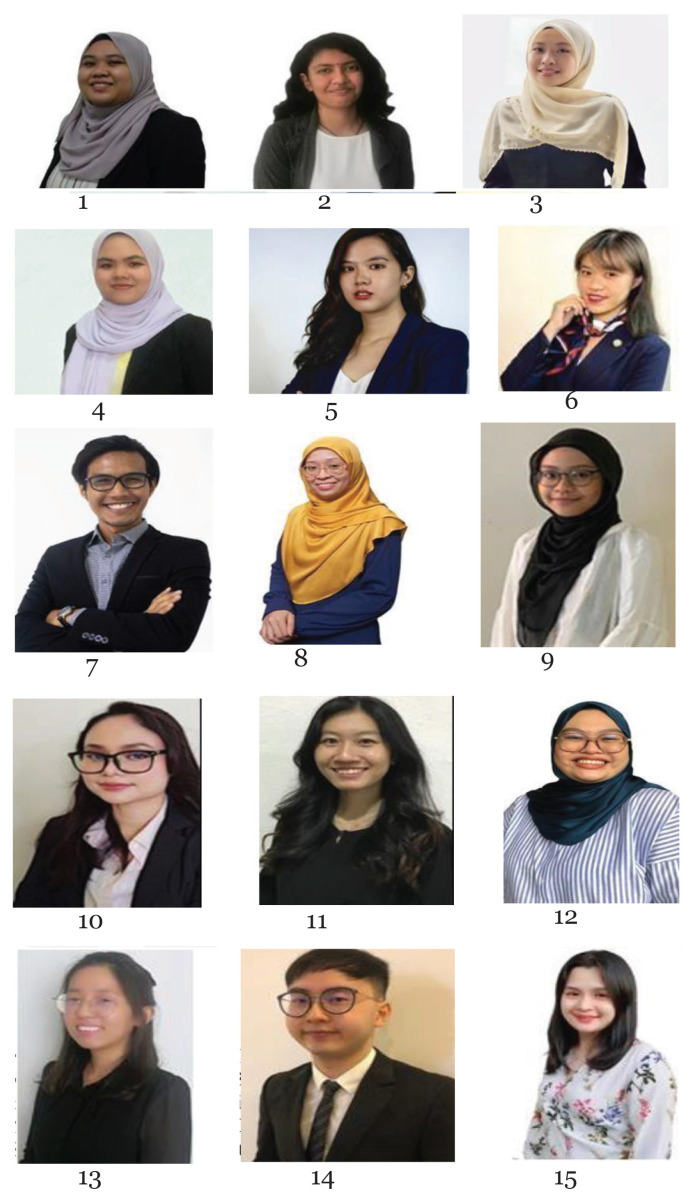
Master of Psychology (Clinical) students (4th batch): (1) Aini Zakirah Zaidi (UPSI), (2) Aishwarya a/p Sivakumaru (UPSI), (3) Aqidah Mazlan (USM), (4) Deanna Arissa Mohd Osman (USM), (5) Fung Chai Ying (USM), (6) Lai Siew Tim (UPSI), (7) Nabil Nasran Nazri (UPSI), (8) Najihah Rosmi (USM), (9) Nurerina Hanizam (UPSI), (10) Nurizzati Saring (UPSI), (11) Pricilla Chuah Ning (USM), (12) Siti Nur Sarah Samberi (USM), (13) Loh Hui Tian (UPSI), (14) Wong Chen Sung (USM) and (15) Leong Mei San (UPSI)

**Figure 10:**
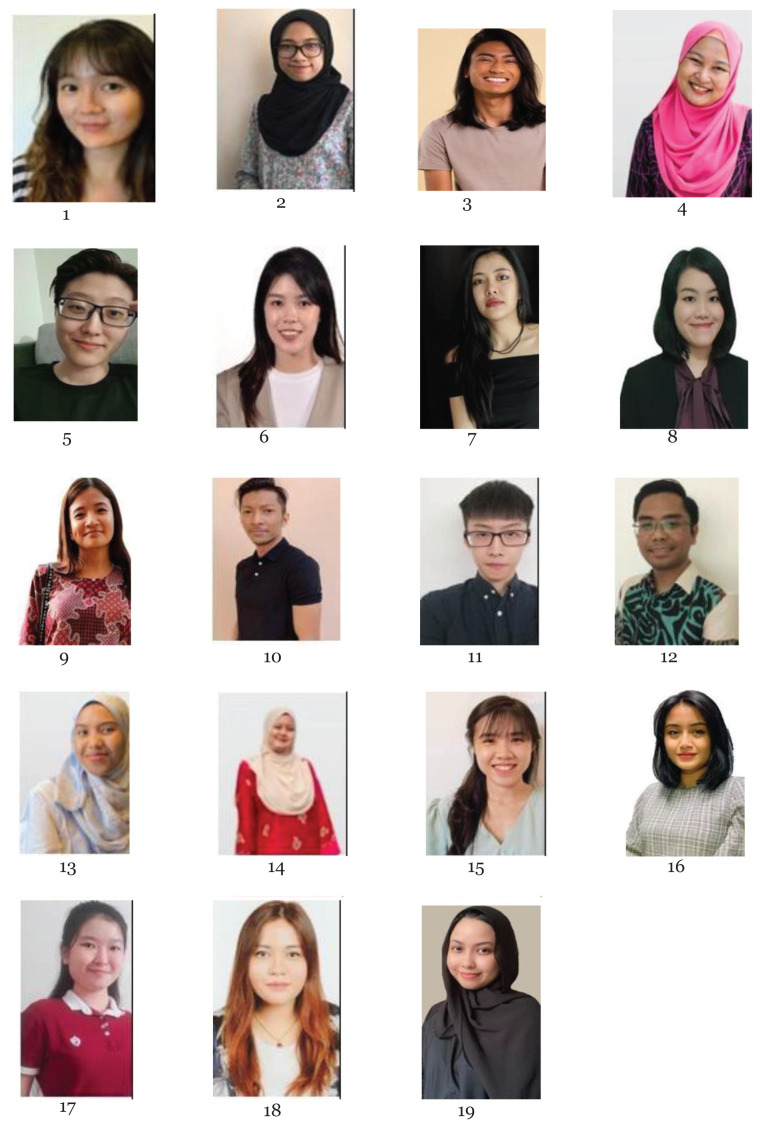
Master of Psychology (Clinical) students (3rd batch) : (1) Alicia Ng Cher Ching (USM), (2) Amirah Zulaikha (USM), (3) Arman bin Imran Ashok (UPSI), (4) Aunei Anuar (UPSI), (5) Chan Kah Mun (UPSI), (6) Chang Kai Ru (UPSI), (7) Chin Yeng Zien (USM), (8) Chong Shao Yin (USM), (9) Ifyani Hazirah (USM), (10) Khair Benjamin bin Lokman (USM), (11) Lee Sheng Kwong (UPSI), (12) Megat Syaiful Izzuddin bin Megat Mokhtar (UPSI), (13) Mumtazah Afifah Abdul Halim (USM), (14) Nur Hafizah Zainol (UPSI), (15) Phoon Ju Yee (UPSI), (16) Syamimi binti Amiruddin (UPSI), (17) Tan Jiun Ting (UPSI), (18) Wan Adibah Nadiah Abd Razak (USM) and (19) Wan Farah Adilah (USM)

**Figure 11:**
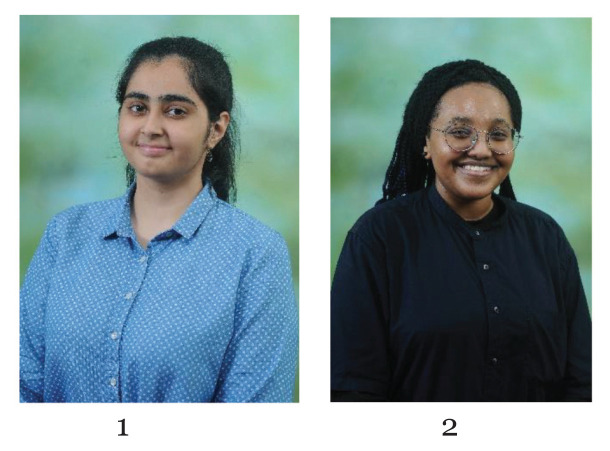
Master of Integrated Neuroscience students: (1) Danniya Lakshmi a/p Manickam and (2) Renad Sadig Muhammed Abdala

**Figure 12:**
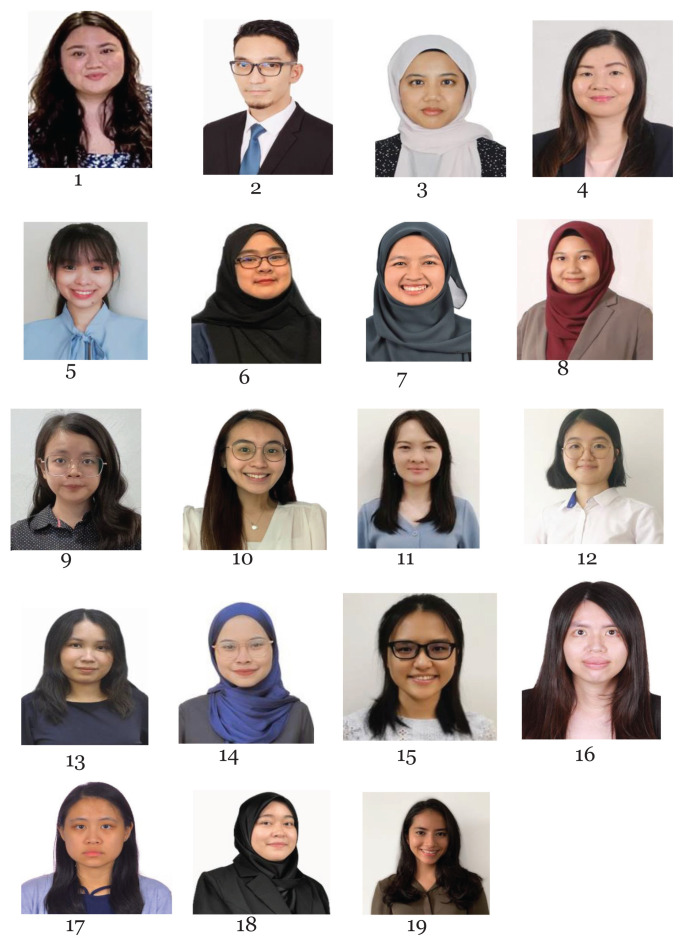
Master of Psychology (Clinical) (5th batch) students, academic session 2022/2023: (1) Jasmine Elenore binti Amir Parrot (USM), (2) Mohd Ashrawi bin Mohd Radzali (USM), (3) Farah Umairah Sallehudin (UPSI), (4) Kuay Hue San (USM), (5) Chong Zi En (UPSI), (6) Nik Hafizah binti Zuraidi Afandi (USM), (7) Nur Shafiqah binti Noor Ashani (USM), (8) Siti Khadijah binti Asha’ari (USM), (9) Esther Goh Xin (UPSI), (10) Ho Yen Ling (UPSI), (11) Ng Lay Hong (USM), (12) Lim Zhi Ying (USM), (13) Lidiya Batrisyia Aderus (UPSI), (14) Dayang Nursyazana (USM), (15) Choy Mun Hui (UPSI), (16) Tan Saw Yen (USM), (17) Cheong Jean Yi (USM), (18) Aisyah Zulkipli (USM) and (19) Audrey Nathan Arul (USM)

**Figure 13:**
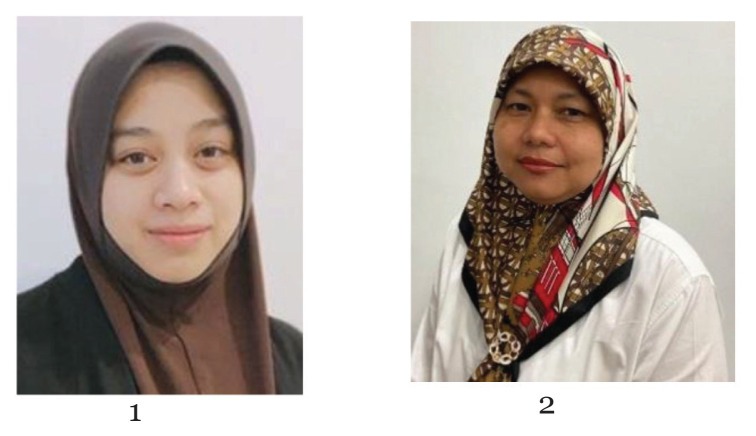
Doctor of Psychology (Clinical Psychology) students, (3rd batch) academic session 2022/2023: (1) Halimatun Syakirah Omar and (2) Zulia Khamis

**Figure 14:**
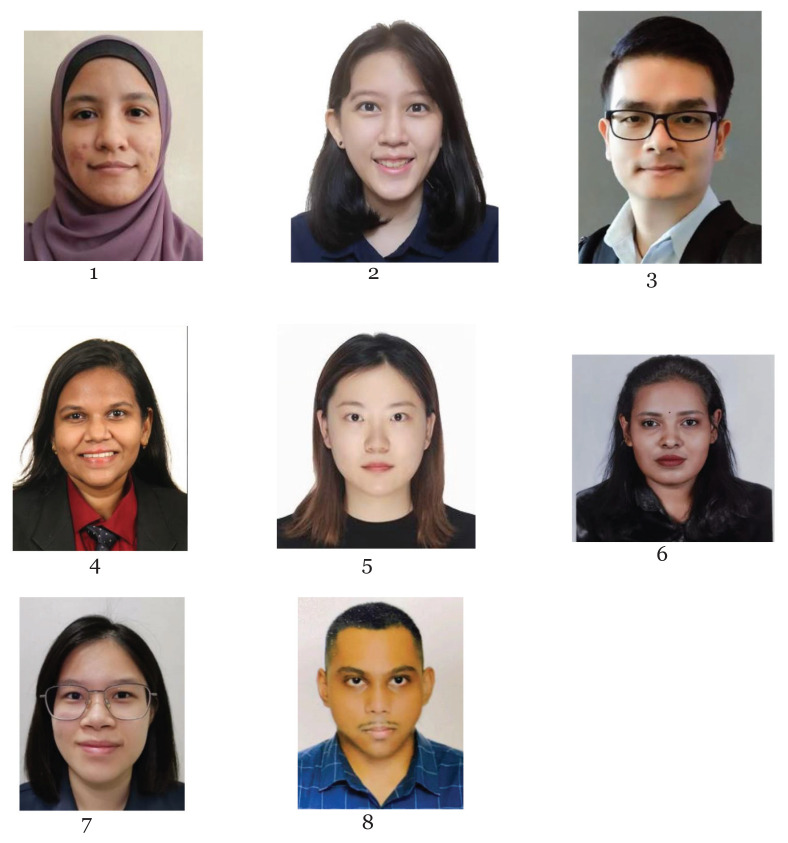
Master of Cognitive Neurosciences students, academic session 2022/2023: (1) Nurul Nadhihah Kamarulzaman, (2) Sin Jo Yee, (3) Tan Lai Soon, (4) Varshini T. Manimudi, (5) Yang Dan, (6) Yoghaanjaly a/p Murugiah, (7) Yoong Weng Kei and (8) Kong Yoke Loong

**Figure 15:**
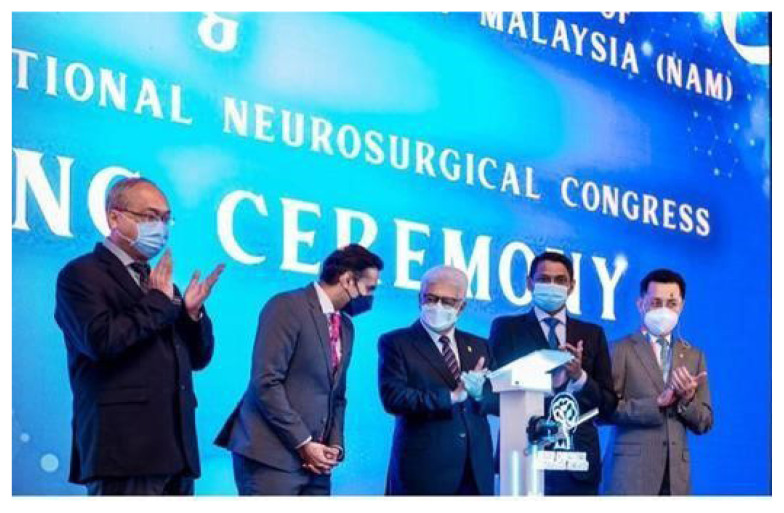
Officiating ceremony of the 20th annual scientific meeting of the Neurosurgical Association of Malaysia: L–R: YBhg Dato’ Dr Maarof Sudin (Pulau Pinang State Health Director), Dr Azman Raffiq (Organising Chairman, Head of Department Neurosurgery Hospital Pulau Pinang), Tuan Yang Terutama Tun Dato’ Seri Utama Ahmad Fuzi bin Haji Abdul Razak, YBhg Dato’ Dr Kantha Rasalingam (President of the Neurosurgical Association of Malaysia) and YBhg Dato’ Dr Azmi Kass Rosman, Head of Neurosurgical Services, Ministry of Health Malaysia

**Figure 16:**
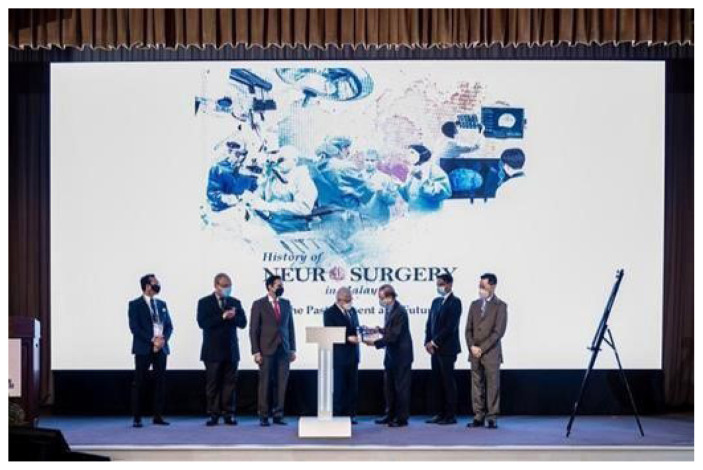
YBhg Dato’ Dr Fazli Cheah presents the book *History of neurosurgery in Malaysia* to Tuan Yang Terutama Tun Dato Seri Utama Ahmad Fuzi bin Haji Abdul Razak as a special gift after the book launch
